# Standardized ileal amino acid digestibility of high-oleic full-fat soybean meal in broilers^1^

**DOI:** 10.1016/j.psj.2023.103152

**Published:** 2023-09-27

**Authors:** Muhammad Ali, Michael Joseph, Maria Camila Alfaro-Wisaquillo, Gustavo Adolfo Quintana-Ospina, Danny Patiño, Lina-Maria Peñuela-Sierra, Thien Vu, Rouf Mian, Earl Taliercio, Ondulla Toomer, Edgar Orlando Oviedo-Rondón

**Affiliations:** ⁎ Prestage Department of Poultry Science, North Carolina State University, Raleigh, NC 27695, USA; † College of Veterinary Medicine and Zootecnia, University of Tolima, Ibagué, Tolima, Colombia; ‡ Trouw Nutrition-Latin America, Ciudad de Guatemala, Guatemala; § Food Science & Market Quality and Handling Research Unit, ARS, USDA, Raleigh, NC 27695, USA; # Soybean and Nitrogen Fixation Research Unit, ARS, USDA, Raleigh, NC 27695, USA

**Keywords:** amino acid digestibility, broiler, high-oleic soybean, full-fat soybeans, soybean meal

## Abstract

High-oleic (**HO**) soybean may serve as a value-added feed ingredient to enrich poultry meat due to its fatty acid content. However, the amino acid (**AA**) nutrient digestibility of soybean meal (**SBM**) made from these soybeans has yet to be determined. The objective of this study was to determine apparent ileal AA digestibility (**AID**) and standardized ileal AA digestibility (**SID**) of high-oleic full-fat (**HO-FF**) SBM compared to normal oleic full-fat (**NO-FF**), normal oleic extruded expeller (**NO-EE**), and solvent-extracted SBM (**SE-SBM**) in broilers. A nitrogen-free basal diet (**NFD**) was fed to 1 treatment group with 10 chicks/cage to determine basal endogenous losses (**BEL**). Titanium dioxide was used as an inert marker. The test diets contained 57.5% of the basal NFD and 42.5% of 1 of the 4 soybean sources. A total of 272 Ross-708 male broilers were placed in 40 battery cages with 5 treatments and 8 replicates per treatment. A common starter diet was provided to all the chickens for 14 d. Experimental diets were provided as a mash for 9 d before sample collection. Chickens were euthanized with CO_2_ on d 23, and contents of the distal ileum were collected, frozen, and freeze-dried. The BEL were similar to the values found in the literature. At d 23, broilers fed the SE-SBM had the highest body weight gain and best FCR compared to chickens fed the HO-FF and NO-FF treatments (*P* < 0.001). Broilers fed the SE-SBM and NO-EE experimental diets had (*P* < 0.001) higher apparent ileal AA digestibility and AA SID than broilers fed the HO-FF and NO-FF treatments. In conclusion, the SID of AA from HO-FF is similar to the digestibilities of other full-fat soybeans found in the literature and is lower than that of NO-EE and SE-SBM.

## INTRODUCTION

Worldwide, corn-soybean diets are the basis of conventional feed for broiler chickens. In broilers, most digestion and absorption of protein and amino acids (**AA**) occur before the digesta transit to the hindgut. Hence, quantifying AA digestibility is essential for precise feed formulation and sustainable feed usage. Digestibility is defined as the total nutrients absorbed. It is calculated as the difference in the amount of incoming nutrients in feed consumed and the amount of nutrients retained in the excreta (Blok et al., 2017), and it is essential to determine the quality of ingested nutrients. Dietary proteins and free AA that are not digested or absorbed in the small intestine (duodenum, jejunum, ileum), in transit to the lower gut, undergo fermentation by bacteria in the caeca and colon. During bacterial fermentation of dietary proteins in the hindgut of broilers, AA may be produced, which cannot be utilized by the host as nutritionally available AAs. Hence, the AAs present within the ileal digesta provide a better assessment of the AA digestibility of a particular feedstuff than those present within the digesta at the end of the digestive tract. Due to this reason, the digestibility of protein and AA at the ileal portion is a vital characteristic of the nutritional value of feed ingredients (Blok et al., 2017), and digestibility in the terminal ileum provides a better appraisal of protein and AA availability than fecal digestibility (Ravindran and Bryden, 1999; Kadim et al., 2002).

Digestibility can be expressed as apparent ileal digestibility (**AID**) or standardized ileal digestibility (**SID**). Apparent ileal digestibility is calculated as the difference between the AA consumed in the feed and the AA recovered from digesta in the distal ileum. In AID, basal endogenous losses (**BEL**) are not considered, while in SID, BEL are calculated and corrected from the AID values (Kadim et al., 2002). The BEL are inevitable losses directly related to the metabolic functions of the animal and not dependent on the diet type (Cowieson et al., 2009). They comprise digestive secretions, mucoproteins, and shed enterocytes (Ravindran, 2021). In broilers, feed ingredient-specific factors can affect the AA SID (Park et al., 2020). Factors like feedstuff, type of protein, crude fiber, and antinutritional factors are essential for determining AA digestibility. Amino acid AID in chickens can also be affected by age (Johns et al., 1986; Huang et al., 2005; An and Kong, 2022) and strain of poultry (Huang et al., 2006, 2007).

Full-fat soybean (**FFSB**) is high in protein and energy and is an important feed ingredient in poultry diets (Ravindran et al., 2014). However, FFSB is used after thermal processing due to the higher concentrations of antinutritional factors, such as trypsin inhibitors (**TI**) in raw whole soybeans (Waldroup, 1982). Protein digestibility and AA availability can be affected by a high level of protease inhibitors present in whole raw SB (Jahanian and Rasouli, 2016). Thermal processing is also applied to SBM after oil extraction from FFSB to inactivate TI and other thermolabile antinutritional factors. However, thermal processing can adversely affect AA digestibility, reducing AA digestibility in FFSB and SBM (Park et al., 2017).

Extrusion processing, followed by mechanical expelling, is a standard method for extracting oil from soybeans and producing extruded expeller SBM (**EE-SBM**) (Bandegan et al., 2010). This method generates a product with higher oil content than solvent-extracted soybean meal (**SE-SBM**) (Webster et al., 2003; Opapeju et al., 2006) but with lower oil than FFSB. Various research groups have also reported differences in AA digestibility coefficients in animals fed EE-SBM compared to SE-SBM (Woodworth et al., 2001; Lawrence et al., 2003; Opapeju et al., 2006; Baker and Stein, 2009).

Today, many soybean-breeding programs have expanded the development of the soybean germplasm to include new high-oleic (**HO**) soybean cultivars with a lipid profile of >75% oleic acid and <1.5% linoleic acid. The oil in these cultivars has improved shelf-life stability, enhanced monounsaturated fatty acid content, and lower saturated fatty acid levels as compared to conventional normal oleic (**NO**) soybean cultivars (30% oleic acid, 7% linoleic acid). However, the AID and SID of these soybean varieties have not been reported. Consequently, the objective of this study is to determine the amino acid AID and SID of the high-oleic full-fat soybean meal (**HO-FF**) compared to SE-SBM, normal oleic extruded expeller (**NO-EE**) SBM, and normal oleic full-fat soybean meal (**NO-FF**) in broilers.

## MATERIALS AND METHODS

All procedures involving the broilers used in this experiment were approved by the North Carolina State University Institutional Animal Care and Use Committee (Approved Protocol # 21-145).

### Meal Preparation and Experimental Diets

Near isogenic lines of conventional NO soybean (<25% oleic acid, >7% linolenic–USDA NC-Roy) and a nongenetically modified HO soybean (>75% oleic acid, <2% linolenic-USDA N16-1286 BC4 NIL) cultivars were bred and harvested by the Soybean and Nitrogen Fixation Research Unit, ARS (Raleigh, NC). Upon harvesting, all foreign material was removed from the soybeans using an Eclipse 324 seed and grain cleaner (Seedburo Equipment Company, Des Plaines, IL), and all whole soybeans were dried to approximately 10% moisture content using cool ambient temperature.

Soybean subsamples were analyzed for mycotoxins (vomitoxin, aflatoxin, fumonisin, ochratoxin, T-2 toxin, zearalenone), trypsin inhibitors, and fatty acid composition by a commercial laboratory using standard methodologies (ATC Scientific, Little Rock, AR). Levels of mycotoxins in the soybean subsamples were below the detection thresholds, and the proximate composition and fatty acid analysis were within the expected parameters. The nutrient and energy content of the soybeans were obtained by near-infrared spectroscopy (**NIRS**) and wet chemistry (Table 1). The NIRS calibration curves used were the AMINONIR RED package (Evonik Animal Nutrition, Hanau-Wolfgang, Germany) for all soybean products (Wiltafsky et al., 2019) that has been evaluated globally and recently by Hack et al. (2023). Five replicates per soybean source were used to determine NIRS values, and an average value was calculated to represent each source.

**Table 1:** Nutrient composition of soybean meal sources used in broiler experimental diets.

Nutrient	SE SBM	NO-EE	NO-FF	HO-FF
Protein, crude, %	47.15	43.80	38.31	38.18
Fat, crude, %	2.57	8.99	18.21	18.21
Fiber, crude, %	3.77	5.27	6.10	6.10
Ash, %	6.83	6.41	5.52	5.52
Calcium, %	0.34	0.20	0.28	0.28
Phosphorus. total, %	0.63	0.57	0.48	0.48
Phosphorus available, %	0.23	0.19	0.16	0.16
Lysine, %	2.83	2.66	2.34	2.40
TSAA, %	0.64	1.21	1.06	1.13
Threonine, %	1.81	1.68	1.49	1.49
Valine, %	2.22	2.07	1.82	1.82
Leucine, %	3.52	3.33	2.86	2.80
Tryptophan, %	1.29	0.59	0.51	0.50
Trypsin inhibitor, mg/g	0.95	7.46	11.02	11.02
Dig. lysine, %	2.57	2.36	2.03	2.09
Dig. methionine, %	0.56	0.51	0.44	0.44
Dig. cystine, %	0.55	0.49	0.40	0.45
Dig. TSAA, %	1.12	1.00	0.84	0.89
Dig. threonine, %	1.52	1.41	1.24	1.24
Dig. tryptophan, %	0.57	0.52	0.43	0.41
Dig. isoleucine, %	1.93	1.82	1.51	1.49
Dig. leucine, %	3.20	2.97	2.50	2.45
Dig. valine, %	1.97	1.80	1.55	1.55
Dig. histidine, %	1.08	0.99	0.87	0.88
Dig. arginine, %	3.20	2.91	2.48	2.58
Dig. phenylalanine, %	2.16	1.96	1.69	1.68
Palmitic acid C16:0, %	0.67	0.80	1.93	1.20
Palmitoleic acid C16, %	0.00	0.01	0.03	0.02
Stearic acid C18:0, %	0.17	0.26	0.61	0.48
Oleic acid C18:1, %	0.68	1.40	3.12	11.13
Linoleic acid C18:2, %	2.65	3.79	9.55	1.71
Linolenic acid C18:3, %	0.41	0.59	1.45	0.02

Abbreviations: HO, high oleic; NO, normal oleic; NO-EE SB, normal oleic extruded expeller soybean; SE-SBM, solvent-extracted soybean meal; TSAA, total sulfur amino acids.

The proximate composition was performed by an AOAC-certified lab, ATC Scientific (Little Rock, AR).

A single screw dry extrusion (InstaPro 2000 R, Grimes, Iowa) process at a die temperature of 155°C was used to produce the NO-FF, HO-FF, and NO-EE SBM at a commercial feed mill, Mule City Feeds (Benson, NC). During this process, a portion of the extruded soybeans were mechanically pressed to extract the oil, and the resulting product had less oil than extruded soybeans. Particle size was homogenized by roller-mill (Model C128889, RMS, Sea, SD) to obtain a geometric mean of 915 to 950 μm. The 50:50 and 50:25 roller gap settings were used for FFSB and EE soybeans, respectively.

Phytase was added to all experimental diets. A starter diet (1–14 d) was crumbled and pelleted at 85°C with a corn particle size of 700 to 800 µm (Table 2). The starter diet containing all soybean sources evaluated in this experiment was formulated using Concept 5 software (Alpharetta, GA) to meet or exceed nutrient requirements for broilers (Ross-708). A nitrogen-free diet (**NFD**) was formulated using corn starch and dextrose sugar (Table 3). Additionally, Solka floc 40 was included as a source of fiber, and all test diets were balanced to meet Ross-708 broiler recommendations. Titanium dioxide was added at 5 g per kg as an inert marker in each diet. Four soybean products were combined with the NFD to create experimental diets. These products were: SE-SBM, NO-EE, NO-FF, and HO-FF. The test diets contained 57.5% basal NFD and 42.5% of the respective SBM source.

**Table 2:** Ingredient and nutrient composition of the broiler experimental starter diet (1–14 d).

Ingredients %	Diet	Nutrient name	Diet
Corn	50.94	M.E. Poultry, kcal/kg	3,000
Solvent-extracted SBM	19.07	Protein crude, %	22.83
Extruded expeller SB	7.00	Fat crude, %	7.33
NO full-fat SB	7.00	Fiber, crude, %	2.93
HO full-fat SB	6.54	Calcium, %	0.94
DDGS	3.75	Phosphorus total, %	0.56
Poultry fat	1.33	Phosphorus available, %	0.34
Limestone fine	1.39	Lysine, %	1.41
Dicalcium phosphate	1.09	TSAA, %	1.05
DL-Methionine	0.37	Threonine, %	1.00
Sodium bicarbonate	0.27	Valine, %	1.05
L-Lysine	0.27	Leucine, %	1.90
Salt, plain (NaCl)	0.28	Tryptophan, %	0.27
Mineral premix^1^	0.20	Trypsin inhibitor, mg/g	2.20
Choline chloride 60	0.18	Dig. lysine, %	1.28
L-Threonine	0.16	Dig. methionine, %	0.66
Vitamin premix^2^	0.10	Dig. cystine, %	0.29
Coccidiostat^3^	0.05	Dig. TSAA, %	0.95
Phytase^4^	0.02	Dig. threonine, %	0.86
		Dig. tryptophan, %	0.23
		Dig. isoleucine, %	0.86
		Dig. leucine, %	1.82
		Dig. valine, %	0.95
		Dig. histidine, %	0.50
		Dig. arginine, %	1.37
		Dig. phenylalanine, %	1.04
		Palmitic acid C16:0, %	0.40
		Palmitoleic acid C16, %	0.01
		Stearic acid C18:0, %	0.12
		Oleic acid C18:1, %	1.17
		Linoleic acid C18:2, %	1.55
		Linolenic acid C18:3, %	0.25

The proximate composition was performed by an AOAC-certified lab, ATC Scientific (Little Rock, AR).

Abbreviations: DDGS, distiller's dried grains with solubles; ME, metabolizable energy; SB, soybean; SBM, soybean meal; TSAA, total sulfur amino acids.

1 Trace minerals provided per kg of premix: manganese (MnSO_4_), 60 g; zinc (ZnSO_4_), 60 g; iron (FeSO_4_), 40 g; copper (CuSO_4_), 5 g; iodine (Ca(IO_3_)_2_), 1.25 g.

2 Vitamins provided per kg of premix: vitamin A, 13,227,513 IU; vitamin D3, 3,968,253 IU; vitamin E, 66,137 IU; vitamin B12, 39.6 mg; riboflavin, 13,227 mg; niacin, 110,229 mg; D-pantothenic acid, 22,045 mg; menadione, 3,968 mg; folic acid, 2,204 mg; vitamin B6, 7,936 mg; thiamine, 3,968 mg; biotin, 253.5 mg.

3 Coban 90 (Monensin), Elanco Animal Health, Greenfield, IN, at 500 g/ton.

4 Natuphos E (500 FTU/kg, 50 g/ton FTU).

**Table 3:** Ingredient and nutrient composition of broiler grower experimental diets (15–23 d).

Ingredients (%)	NFD	Test diets			
		SE SBM	NO-EE	NO-FF	HO-FF
Corn starch	78.08	40.26	43.5	45.13	45.13
Dextrose sugar	6.50	6.50	6.50	6.50	6.50
Soybean oil	5.00	4.00	1.21		
Solka floc 40^1^	3.00	1.36	1.42	1.06	1.06
Dicalcium phosphate	2.38	1.84	1.95	2.00	2.00
Limestone fine	0.95	0.86	0.96	0.84	0.84
Titanium dioxide	0.50	0.50	0.50	0.50	0.50
Salt	0.36	0.33	0.34	0.32	0.32
Sodium bicarbonate	0.05	0.14	0.10	0.10	0.10
Mineral premix^2^	0.20	0.20	0.20	0.20	0.20
Choline chloride	0.30	0.30	0.30	0.30	0.30
Vitamin premix^3^	0.10	0.10	0.10	0.10	0.10
Soybean meal solvent extracted		42.50			
Extruded expeller soybean			42.50		
Normal oleic full-fat soybean				42.50	
High-oleic full-fat soybean					42.50
Potassium carbonate	1.33	0.05	0.06	0.05	0.05
Arbocel^1^	1.00	1.00	0.30	0.30	0.30
Magnesium oxide 58%	0.26	0.06	0.08	0.09	0.09
	100	100	100	100	100
Nutrient					
M.E. poultry, kcal/kg	3,638	3,136	3,153	3,360	3,360
Dry matter, %	90.63	90.39	91.96	92.07	92.07
Protein, crude, %	0.07	20.07	18.65	16.32	16.27
Fat, crude, %	5.11	5.13	5.11	7.83	7.83
Fiber, crude, %	4.08	4.00	4.00	4.00	4.00
Calcium, %	0.87	0.87	0.87	0.87	0.87
Phosphorus available, %	0.44	0.44	0.44	0.44	0.44
Sodium, %	0.20	0.20	0.20	0.20	0.20
Potassium, %	0.76	0.93	0.76	0.76	0.76
Chloride, %	0.25	0.25	0.25	0.25	0.25
Trypsin inhibitor, mg/g		0.40	3.17	4.68	4.68
Dig. lysine, %		1.09	1.00	0.86	0.89
Dig. methionine, %		0.24	0.22	0.19	0.19
Dig. cysteine, %		0.24	0.21	0.17	0.19
Dig. total sulfur amino acids, %		0.47	0.43	0.36	0.38
Dig. threonine, %		0.65	0.60	0.53	0.53
Dig. tryptophan, %		0.24	0.22	0.18	0.18
Dig. isoleucine, %		0.82	0.77	0.64	0.63
Dig. leucine, %		1.36	1.26	1.06	1.04
Dig. valine, %		0.84	0.77	0.66	0.66
Dig. arginine, %		1.36	1.24	1.05	1.10
Dietary electrolytes, MEQ/kg	225	269	225	225	225
Palmitoleic acid C:16, %		0.00	0.00	0.01	0.01
Stearic acid C18:0, %		0.07	0.11	0.26	0.20
Oleic acid C18:1, %		0.29	0.60	1.33	4.73
Linoleic acid C18:2, %		1.13	1.61	4.06	0.73
Linolenic acid C18:3, %		0.17	0.25	0.62	0.21

Abbreviations: HO, high oleic; ME, metabolizable energy; NFD, nitrogen-free diet; NO, normal oleic; NO-EE SB, normal oleic extruded expeller soybean; SE-SBM, solvent-extracted soybean meal.

The proximate analysis was conducted by an AOAC-certified lab, ATC Scientific (Little Rock, AR).

1 Sources of purified cellulose and other insoluble fibers to provide similar dietary fiber in nitrogen-free and test diets.

2 Trace minerals provided per kg of premix: manganese (MnSO_4_), 60 g; zinc (ZnSO_4_), 60 g; iron (FeSO_4_), 40 g; copper (CuSO_4_), 5 g; iodine (Ca(IO_3_)_2_), 1.25 g.

3 Vitamins provided per kg of premix: vitamin A, 13,227,513 IU; vitamin D3, 3,968,253 IU; vitamin E, 66,137 IU; vitamin B12, 39.6 mg; riboflavin, 13,227 mg; niacin, 110,229 mg; d-pantothenic acid, 22,045 mg; menadione, 3,968 mg; folic acid, 2,204 mg; vitamin B6, 7,936 mg; thiamine, 3,968 mg; biotin, 253.5 mg.

### Chicken Husbandry and Sample Collection

A total of 272 Ross-708 broiler chicks were used in this experiment. Male chickens were individually weighed, tagged, and separated by feather sexing to reduce the data variation. Chicks were randomly placed in battery cages. Brooder battery cages (88 × 32 × 24 cm^3^) were electrically heated, and the room temperature was maintained at 32°C, 28°C, and 24°C during wk 1, 2, and 3, respectively, under a 23 h light and 1 h dark cycle. Mortality was recorded daily. Each treatment had 8 replicate cages. The same starter diet with 3,000 kcal/kg energy and 22% crude protein (**CP**) was provided to all the chicks until the birds were 14-day old. Experimental diets were provided during the next 9 d before sample collection. Feed and water were provided to the broilers unrestricted for the whole experimental period. The NFD had 10 broilers per cage to collect enough ileal digesta samples to be analyzed, while all others had 6 broilers per cage. NFD was fed to determine basal endogenous losses. Chickens were euthanized with CO_2_ at d 23, and the distal ileum (lower half) contents were collected by gently flushing sections of the distal ileum with distilled water. The ileum was defined as that portion of the small intestine extending from the vitelline diverticulum (formerly Meckel's diverticulum) to a point 40 mm proximal to the ileo-cecal junction. Collected ileal contents of all chickens per pen were then pooled, frozen, stored at −20°C, and freeze-dried before lab analysis.

### Chemical Analysis

Feed and ileal samples were ground finely using a coffee grinder and analyzed for dry matter (**DM**), CP, AA, and titanium dioxide contents. The DM of feed and ileal samples were evaluated by drying in a forced draft oven (NFTA Method 2.1.4, AOAC Official Method 935.29 & 945.15). Ground feed and digesta samples were analyzed by the University of Missouri Experiment Station and Chemical Laboratories (Columbia, MO) for AA levels by high-performance liquid chromatography [Method 982.30 E (a, b, c), AOAC International, 2006]. Titanium dioxide concentration was measured in triplicate for feed samples and duplicate for ileal samples using a UV spectrophotometer following methods described by Myers et al. (2004).

### Digestibility Calculations

Ileal endogenous AA losses in broilers fed the NFD were calculated as milligrams of AA flow per 1 kg of DM intake (**DMI**) using the following formula by Moughan et al. (1992):

(1) IlealAAlosses,mg/kgofDMI=[AAinilealdigesta,mg/kg×(diettitanium,mg/kg/ilealtitanium,mg/kg)]

(2) ApparentilealAAdigestibility,%=[1−(titaniumindiet/titaniuminilealdigesta)×(AAindigesta/AAindiet)]

(3) StandardilealAAdigestibility(SID),%=Apparentdigestibility,%+[(endogenouslossesAA,g/kgofDMI)/(AAcontentoftherawmaterial,g/kgofDM)]×100

### Experimental Design and Statistical Analysis

Data were analyzed using JMP Pro 15 software (SAS Institute, Inc., Cary, NC). Cage served as the experimental unit. Prior to statistical analyses, the distribution platform of JMP was used to verify normality. Any outliers, determined as 3 times the root mean square error (**RMSE**) plus or minus the mean of the response, were removed from the statistical analysis.

Data were analyzed in a completely randomized design using ANOVA. The AID and SID values for each SBM source were presented as the least squares mean. Mean separation was assessed using Tukey's or Student *t* test at the significance level of *P* < 0.05.

## RESULTS

The nutrient content of the 4 soybean meal sources used in the preparation of the experimental diets is presented in Table 1. In contrast, the ingredient list and nutrient composition of the experimental diets are represented in Table 3. Apparent ileal AA digestibility was obtained from each of the 4 diets containing the soybean meals, and BEL was obtained from the NFD to calculate SID. Broiler live weights and feed intake were determined on d 14, 19, and 23 (Table 4). There were no significant treatment differences in any of the production parameters (BW, BW gain, FCR, feed intake) measured between d 0 to 14 (*P* > 0.05). However, on d 23, broilers fed the SE-SBM had the highest BW and BW gain (*P* < 0.001) compared to the other treatments. Broilers fed the SE-SBM had better FCR (*P* < 0.001) than broilers fed the HO-FF, NO-FF, and NFD dietary treatments. The FCR of chickens fed SE-SBM was similar to that observed in the group fed the NO-EE diet. Between the 14- to 23-day period and at 23 d, broilers fed the NFD experimental diet had lower (*P* < 0.001) feed intake relative to the other treatment groups. In contrast, feed intake was similar between broilers fed the SE SBM, NO-EE, NO-FF, and HO-FF treatments. The FCR was similar between broilers fed the NO-FF, HO-FF, NO-EE, and SE SBM diets during the 14- to 23-day period, while FCR was significantly higher (*P* < 0.001) and as expected adversely affected in broilers fed the NFD diet. Furthermore, chickens fed the SE-SBM diet exhibited the best BW gain, and the NFD broilers had the lowest BW gain relative to the other treatment groups during the 14- to 23-day period, with similar BW gain in the NO-EE and HO-FF treatment groups (*P* < 0.001). However, broilers raised on NFD diets had the lowest feed intake (*P* < 0.001) and lost BW compared to other treatments.

**Table 4:** Live performance of Ross-708 male broilers in battery cages before and after the experimental period according to the assigned dietary treatment.

Live performance	NFD	Solvent-extracted SBM	Extruded expeller soybean	Full-fat		SEM	CV%	*P* value
				Normal oleic	High oleic			
0–14 d								
BW, g	497	483	477	489	505	7.80	4.30	0.108
BWG, g	454	439	433	445	461	7.72	4.68	0.098
Feed intake, g	515	499	501	499	514	7.26	3.93	0.297
FCR (g:g)	1.136	1.130	1.134	1.122	1.117	0.01	2.22	0.527
0–23 d								
BW, g	442^c^	924^a^	844^b^	794^b^	847^b^	15.05	5.48	<0.001
BWG, g	398^c^	879^a^	800^b^	750^b^	803^b^	15.02	5.80	<0.001
Feed intake, g	1,097^b^	1,321^a^	1,293^a^	1,295^a^	1,331^a^	22.88	4.96	<0.001
FCR (g:g)	2.757^a^	1.534^b^	1.598^cd^	1.726^d^	1.660^bc^	0.02	2.97	<0.001
14 - 23 d								
BWG, g	−59^d^	421^a^	353^b^	296^c^	330^bc^	12.3	12.96	<0.001
Feed intake, g	582^b^	825^a^	792^a^	795^a^	817^a^	18.4	6.82	<0.001
FCR (g:g)	10.232^b^	1.974^a^	2.258^a^	2.712^a^	2.480^a^	0.32	566.80	<0.001

Abbreviations: BW, body weight; BWG, body weight gain; FCR, feed conversion ratio; NFD, nitrogen-free diet; SBM, soybean meal.

a–d Means in a column not sharing a common superscript are significantly different (*P* < 0.001) by Student *t* or Tukey's test.

The BEL (g/kg per DMI) and coefficient of apparent digestibility of DM (**CADDM**) were obtained and presented in Table 5. The AA Leu and Glu contributed the highest amounts to BEL for indispensable and dispensable AA, respectively. BEL of Trp for indispensable AA and Cys for dispensable AA were observed to be the lowest values. Broilers fed the SE SBM treatment group had the highest CADDM relative to the other treatment groups (*P* < 0.001), with similar CADDM values between the NO-EE, HO-FF, and NO-FF treatment groups. Broilers fed the SE SBM and NO-EE experimental diets had significantly higher apparent ileal CP and total AA digestibility values than those found in broilers fed the HO-FF and NO-FF treatments (*P* < 0.001). In contrast, values were similar between the NO-FF and HO-FF treatment groups.

**Table 5:** Apparent ileal (AID) amino acid digestibility of feed ingredients and coefficient of apparent digestibility of dry matter (CADDM) of experimental broiler diets.

				Full-fat				
Items	Basal endogenous loses	Solvent-extracted SBM	Extruded expeller soybean	Normal oleic	High oleic	SEM	CV %	*P* value
	g/kg DMI	%						
CADDM		48.81^a^	40.48^b^	36.65^b^	40.37^b^	1.37	9.15	<0.001
Indispensable amino acids								
Arg	0.27	92.34^a^	91.64^a^	88.24^b^	89.16^b^	0.56	1.74	<0.001
Ile	0.43	85.85^a^	82.71^b^	78.03^c^	76.69^c^	0.76	2.61	<0.001
Leu	0.56	86.03^a^	83.60^a^	79.19^b^	78.88^b^	0.71	0.05	<0.001
Lys	0.38	89.20^a^	87.00^b^	83.81^c^	84.75^c^	0.52	1.67	<0.001
Met	0.12	90.71^a^	87.03^b^	82.72^c^	82.36^c^	0.66	2.10	<0.001
Thr	0.46	82.65^a^	78.00^b^	74.23^c^	75.10^bc^	0.89	3.25	<0.001
Trp	0.07	89.95^a^	84.24^b^	81.77^b^	81.82^b^	0.80	2.67	<0.001
Val	0.34	85.58^a^	82.78^a^	77.98^b^	78.02^b^	0.87	3.04	<0.001
Dispensable amino acids								
Ala,	0.37	85.95^a^	83.48^a^	79.71^b^	78.54^b^	0.67	2.23	<0.001
Asp	0.74	85.22^a^	84.20^a^	80.29^b^	80.93^b^	0.63	2.08	<0.001
Cys	0.23	77.32^a^	69.41^b^	63.27^c^	66.47^bc^	1.12	4.49	<0.001
Glu	0.79	89.61^a^	89.37^a^	86.27^b^	86.87^b^	0.48	1.50	<0.001
Gly	0.40	84.05^a^	78.85^b^	74.64^c^	74.02^c^	0.87	3.17	<0.001
Ser	0.45	85.52^a^	81.15^b^	76.40^c^	75.71^c^	0.80	2.74	<0.001
TAA	8.85	86.57^a^	84.44^a^	79.50^b^	80.12^b^	0.65	2.18	<0.001
CP	12.35	83.13^a^	81.44^a^	76.59^b^	78.11^b^	0.72	2.51	<0.001

Abbreviations: CADDM, coefficient of apparent digestibility of dry matter; CP, crude protein; SBM, soybean meal; TAA, total amino acids.

a–c Means in a column not sharing a common superscript are significantly different (*P* < 0.001) by Student *t* or Tukey's test.

The SE-SBM and NO-EE had the highest AID coefficients (*P* < 0.001) for indispensable (Arg, Ile, Leu, Lys, Met, Thr, Trp, and Val) and dispensable (Ala, Asp, Cys, Glu, Gly, and Ser) AAs. The coefficients of AID for the same indispensable and dispensable AAs were similar between the NO-FF and HO-FF treatment groups. Subsequently, the SID of AA was calculated utilizing the BEL (Table 5) and applying Equation (3), described in the MATERIALS AND METHODS section. In parallel with the AA AID, the highest (*P* < 0.001) AA SID of the indispensable and dispensable AAs was observed in the SE-SBM and NO-EE treatment groups (Table 6) and the lowest in the NO-FF and HO-FF. The FF products did not differ in AID or SID coefficients for any AA evaluated. Nevertheless, the AID and SID coefficients of Ile, Lys, Met, Thr, Trp, Cys, Gly, and Ser were lower in NO-EE than for SE-SBM.

**Table 6:** Calculated amino acid standardized ileal digestibility (SID) of soybean sources^1^.

			Full-fat		
Items	Solvent-extracted SBM	Extruded expeller soybean meal	Normal oleic	High oleic	*P* value
	%				
Indispensable amino acids					
Arg	94.16^a^	93.67^a^	90.60^b^	91.29^b^	<0.001
Ile	89.87^a^	87.41^b^	82.68^c^	82.05^c^	<0.001
Leu	89.36^a^	87.42^a^	82.94^b^	83.18^b^	<0.001
Lys	91.93^a^	90.66^b^	87.45^c^	88.10^c^	<0.001
Met	94.73^a^	91.22^b^	87.08^c^	87.60^c^	<0.001
Thr	87.90^a^	84.11^b^	81.27^c^	81.73^bc^	<0.001
Trp	92.37^a^	87.19^b^	84.85^b^	85.07^b^	<0.001
Val	88.76^a^	86.42^a^	82.14^b^	82.02^b^	<0.001
Dispensable amino acids					
Ala	89.69^a^	87.18^a^	84.04^b^	83.42^b^	<0.001
Asp	88.70^a^	88.19^a^	84.26^b^	84.72^b^	<0.001
Cys	84.49^a^	77.44^b^	71.43^c^	74.41^bc^	<0.001
Glu	92.00^a^	92.11^a^	88.92^b^	89.43^b^	<0.001
Gly	88.20^a^	83.71^b^	80.20^c^	79.36^c^	<0.001
Ser	90.09^a^	86.94^b^	82.44^c^	80.11^c^	<0.001
TAA	90.63^a^	88.65^a^	84.96^b^	85.29^b^	<0.001
CP	89.03^a^	87.37^a^	84.03^b^	84.95^b^	<0.001

1 Values calculated as the following: Amino acid standardized ileal digestibility (SID), % = Apparent digestibility, % + [(indigenous losses AA, g/kg of DMI)/(AA content of the raw material, g/kg of DM)] x 100. Abbreviations: CP, crude protein; SBM, soybean meal; TAA, total amino acids.

a–c Means in a column not sharing a common superscript are significantly different (*P* < 0.001) by Student *t* or Tukey's test.

## DISCUSSION

While several studies have examined the ileal AA digestibility coefficients of SE-SBM and FFSB broiler chickens under specific processing conditions of normal oleic soybeans, there is limited available data regarding the ileal AA digestibility of HO-FF for broilers. Valencia et al. (2009) presented the AA SID data of FFSB, but they used micronized FFSB instead of extruded SB. As expected, the CP and AA concentrations were lower in HO-FF and NO-FF than in NO-EE and SE SBM. It is worth noting that both sources of FFSB used in the present experiment underwent exclusive extrusion processes, resulting in the highest oil content. Interestingly, the concentrations of these nutrients were intermediary in the NO-EE SBM sources when compared to FFSB and SE-SBM.

The range of TI values (mg/g) in the experimental broiler diets was 0.40 to 4.68 mg/g. The lowest TI was observed in the SE-SBM and highest in FFSB, with both FFSB sources having the same TI values. The SE-SBM and the NO-EE samples had lower TI levels than the FFSB sources due to the additional heat processing and inactivation of TI during oil extraction in the solvent-extraction and expelling process. Higher TI levels in FFSB can be mitigated during processing with increased temperature and time during conditioning or expansion of FFSB to reduce antinutritional factors (Heger et al., 2016). In light of these findings, it can be inferred that extrusion processing of FFSB alone did not adequately optimize its AA availability while effectively reducing antinutritional factors

The BEL of indispensable and dispensable AA for broiler chickens observed in the current experiment were comparable to previously published studies; however, the BEL of dispensable AA were slightly lower (Kong and Adeola, 2013; Adedokun et al., 2014; Ravindran et al., 2014; Park et al., 2017). The estimation of ileal BEL in broiler chickens has been commonly facilitated by utilizing the NFD in various studies (Adedokun et al., 2008, 2014; Kong and Adeola, 2013; Barua et al., 2020). In these studies, the NFD often contains starch and dextrose. Still, other published digestibility studies have utilized sucrose and corn starch in the NFD to measure BEL (Park et al., 2019, 2020).

Most recent studies (Adedokun et al., 2017; Zhou et al., 2022) have shown that the dietary source of energy (corn starch, dextrose, or sucrose) and their proportions in the NFD directly affect ileal BEL and individual AA digestibility. Zhou et al. (2022) reported that NFD with a dextrose to corn starch ratio between 1.00 and 0.60 increased BEL with enhanced production of digestive enzymes and mucin secretion, while NFD with a dextrose to corn starch ratio at 0.33 promoted a normal digestive physiological state. Hence, this suggests that a NFD with a dextrose to corn starch ratio of 0.33 or lower is most desirable for use in NFD to measure BEL. Nonetheless, there are published AA digestibility studies that have utilized a NFD with a dextrose-to-corn starch ratio between 3.4 and 3.6 (Adedokun et al., 2008, 2014, 2017; Kong and Adeola, 2013; Park et al., 2017) and the sucrose-to-corn starch ratio of 1.58 (Park et al., 2019, 2020). In the experiment described here, the NFD was made with corn starch (78.08%) and dextrose (6.50%) with a dextrose to corn starch ratio 0.08. This ratio is lower than any previously reported in the literature (Adedokun et al., 2008, 2014,2017; Kong and Adeola, 2013; Park et al., 2017) and hence should not have altered the normal physiological state of the ileum. Nonetheless, additional studies are needed to understand better the role of the dietary dextrose to corn starch in NFD on the enteric function in poultry.

In a study conducted by Iyayi and Adeola (2014), the AA SID of FFSB was calculated and compared with other feed ingredients (peanut flour, wheat bran, corn, sorghum, and fishmeal) for broilers at 26 d. For the SID of indispensable AA of FFSB, the highest coefficient observed was for Arg (84.9%), and the lowest was for Thr (69.8%). For the SID of dispensable AA of FFSB, the highest coefficient observed was Glu (84.3%), and the lowest value was for Cys (56.9%). The same AAs had the highest and lowest SID in the present experiment, but our values were higher than reported in previous studies.

The AID and SID coefficients of all AAs were higher in SE-SBM and NO-EE than in NO-FF and HO-FF. This difference has been reported in previous studies. Park et al. (2017) compared the SID coefficients of FFSB with 2 types of SE-SBM (43 and 47% CP) in broilers at 26 d. They demonstrated that the AA SID of FFSB was lower (*P* < 0.05) than that of either SE-SBM (43 or 47%) for all indispensable and dispensable AA. On average, FFSB had 9.49 and 10.50% lower AA SID values than SE SBM. In the present study, the differences in SID digestibility coefficients were between 2.87 and 10.08% points, consistently lower for the FFSB.

In the current experiment, the SID of TAA and CP (Table 6) of the HO-FF were lower than the NO-EE and SE-SBM coefficients. This could be partially due to the higher TI levels in the FFSB sources (4.68% vs. 3.17 and 0.40% in NO-EE and SE SBM, respectively). In a Palliyeguru et al. (2011) study, the AID of CP in broilers fed a corn SBM diet with 300 g/kg FFSB linearly decreased as the TI increased from 3.61 to 16.1 TIU//mg. They concluded that a higher level of TI in soybeans might be the main reason for low CP digestibility. However, in a study by Clarke and Wiseman (2005), a variation was observed in digestible AA content even though the TI values in both samples were very similar (3.6 and 3.4 mg/g). This implies that together with TI, other dietary variables may affect the AA digestibility in FFSB. Valencia et al. (2009) also reported lower values of SID of CP, Leu, Met, Val, and Ala in broilers fed an FFSB or SE-SBM diet.

In the present study, experimental diets were given for 9 d, and digesta samples were collected at 23 d of age. The age of collection and adaptation period may affect the SID values. Recent research (An and Kong, 2022) demonstrated that digestibility (AID or SID) of individual AA in broilers increases as chickens age during the first 3 wk of life, and age-specific AA BEL standardization is necessary. Ravindran et al. (2017) recommended using 21- to 35-day-old chickens and an adaptation period minimum of 5 d before sample collection. We used a more extended adaptation period due to the variability in the fat content of the soybean products evaluated. Adedokun et al. (2017) concluded that differences detected in AA BEL by feeding length are not large enough to influence SIAAD values in 21-day-old chickens.

The differences in the digestibility coefficient values among the various studies may be due to differences in processing methods (Heger et al., 2016), FFSB sources, or both (An et al., 2022). Heating time plays a significant role in the processing of FFSB. The BW gain and feed efficiency of broilers fed diets with 371.8 g/kg FFSB increased quadratically when the autoclaving time of FFSB varied between 0 and 90 min at 121°C. However, chickens fed with FFSB autoclaved for more than 40 min had reduced BW and feed efficiency (Herkelman et al., 1991). Therefore, this factor can lead to differences in AA digestibility.

The gizzard is the main organ in chickens that regulates the passage of digesta to the intestine, and particle size can influence nutrient digestibility. In the current study, the average geometric mean particle size of all soybean sources was uniform between all experimental diets. Another well-known factor that influences digestibility is dietary fiber. In the current study, a fiber content of 4% was kept similar for all the treatments with 2 sources of cellulose and insoluble fiber (Solka floc 40 and Arbocel). However, fat content was different among the treatments. The NO-FF and HO-FF diets did not receive soybean oil, but the HO-FF and NO-FF test diets contained 7.83% fat. In contrast, the NO-EE and SE-SBM test diets had 5.11 and 5.13% fat. The higher fat content in the FFSB test diets could result in lower AA digestibility due to the overall nutrient dilution effect and imbalance between energy and AA supply (Ravindran et al., 2014). All these factors play a role in the values reported here.

Numerous feeding trials have demonstrated the effective utilization of HO oilseeds and FF meals as alternative feed ingredients in poultry production to enrich meat or eggs with oleic acid. Hence, additional studies must be conducted to better define optimal feed processing methods and increase the nutrient digestibility of HO-FF and NO-FF SBM in poultry diets. Using HO soybean cultivars to produce HO-FF SBM could create a potential value-added feed ingredient for the poultry meat and egg production industry, with potential economic benefits for the soybean and poultry industries.

## CONCLUSIONS

The AA AID and SID coefficients of HO-FF soybean meals are similar to those observed in normal oleic full-fat meals obtained by extrusion in broilers. These coefficients are lower than the NO-EE and SE-SBM. The AA BEL determined in this experiment were similar to those reported in the literature for experiments using similar methodologies.
